# Modified Sequential Organ Failure Assessment Score vs. Early Warning Scores in Prehospital Care to Predict Major Adverse Cardiac Events in Acute Cardiovascular Disease

**DOI:** 10.3390/jcdd10020088

**Published:** 2023-02-17

**Authors:** Enrique Castro Portillo, Raúl López-Izquierdo, Miguel A. Castro Villamor, Ancor Sanz-García, José L. Martín-Conty, Begoña Polonio-López, Irene Sánchez-Soberón, Carlos del Pozo Vegas, Carlos Durantez-Fernández, Rosa Conty-Serrano, Francisco Martín-Rodríguez

**Affiliations:** 1 Emergency Department, Hospital Universitario Rio Hortega, 47012 Valladolid, Spain; 2 Faculty of Medicine, Universidad de Valladolid, 47003 Valladolid, Spain; 3 Faculty of Health Sciences, Universidad de Castilla la Mancha, 45600 Talavera de la Reina, Spain; 4 Advanced Life Support, Emergency Medical Services (SACYL), 47007 Valladolid, Spain; 5 Emergency Department, Hospital Clínico Universitario, 47003 Valladolid, Spain; 6 Department of Nursing, Faculty of Nursing, Universidad de Valladolid, 47003 Valladolid, Spain; 7 Faculty of Nursing, Universidad of Castilla-La Mancha, 45004 Toledo, Spain

**Keywords:** biomarker, early warning score, mayor adverse cardiac event, prehospital care, short-term mortality

## Abstract

(1) Background: The Modified Sequential Organ Failure Assessment (mSOFA) is an Early Warning Score (EWS) that has proven to be useful in identifying patients at high risk of mortality in prehospital care. The main objective of this study was to evaluate the predictive validity of prehospital mSOFA in estimating 2- and 90-day mortality (all-cause) in patients with acute cardiovascular diseases (ACVD), and to compare this validity to that of four other widely-used EWS. (2) Methods: We conducted a prospective, observational, multicentric, ambulance-based study in adults with suspected ACVD who were transferred by ambulance to Emergency Departments (ED). The primary outcome was 2- and 90-day mortality (all-cause in- and out-hospital). The discriminative power of the predictive variable was assessed and evaluated by the area under the curve (AUC) of the receiver operating characteristic (ROC). (3) Results: A total of 1540 patients met the inclusion criteria. The 2- and 90-day mortality rates were 5.3% and 12.7%, respectively. The mSOFA showed the highest AUC of all the evaluated scores for both 2- and 90-day mortality, AUC = 0.943 (0.917–0.968) and AUC = 0.874 (0.847–0.902), respectively. (4) Conclusions: The mSOFA is a quick and easy-to-use EWS with an excellent ability to predict mortality at both 2 and 90 days in patients treated for ACVD, and has proved to be superior to the other EWS evaluated in this study.

## 1. Introduction

Rapid and effective identification of high-risk-mortality patients is a major challenge for emergency medical services (EMS). Consequently, several early warning scores (EWS) have been designed, generally based on vital signs and biomarkers, to facilitate the assessment of patients suffering from acute diseases, thus improving management from prehospital care to emergency department (ED) [1,2]. Short-term forecasting can facilitate timely activation of specific treatment codes, allowing better hospital management [3].

Acute cardiovascular disease (ACVD) is one of the commonest reasons for emergency treatment by EMS [4] and is also the leading cause of hospitalization in older adults (patients over 65 years) [5]. Certain EWS are specially tailored to evaluate cardiovascular conditions such as chest pain or non-ST Elevation Myocardial Infarction; however, no standardized scoring system can currently tag high-risk ACVD patients from all-cause mortality in prehospital care [6,7].

Nevertheless, cardiovascular risk stratification is usually performed using risk scores calculated from specific biomarkers (e.g., troponin, myoglobin, creatinine clearance), or using echocardioscopy techniques. Unfortunately, these tests are not available on-scene so the screening of high-risk patients undergoing major adverse cardiac events (MACE) is based on objective and structured clinical evaluation focusing on the exploration of key clinical symptoms (e.g., chest pain, dyspnea, congestion signals), together with standard vital signs (respiratory rate, oxygen saturation, blood pressure, heart rate, temperature, and level of consciousness), electrocardiograms, continuous monitoring, and, in certain EMS, the use of bedside point-of-care testing (POCT).

The Sequential Organ Failure Assessment (SOFA) score is an essential scoring system used by healthcare providers to estimate mortality in critically ill patients and has proven useful in a wide range of clinical situations [8]. However, nowadays, certain analytical variables (platelets and bilirubin) require complex laboratory equipment, making it difficult to perform these tests on-scene or en route. To overcome this problem, a modified SOFA score (mSOFA) has been developed, providing an ability to predict short-term mortality equivalent to SOFA, while being simpler to use. The mSOFA is applicable and adapted to analytical variables provided by the point-of-care testing (POCT) currently available in prehospital care [9,10].

The primary endpoint for this study was to evaluate the predictive validity prehospital of the mSOFA in estimating 2- and 90-day mortality (all-cause) in patients with ACVD, and to compare the results to four EWS: the TIMI risk index (TIMI), the modified shock index (MSI), the Cardiac Arrest Risk Triage (CART) and the National Early Warning Score 2 (NEWS2). In addition, the secondary objective was to evaluate the performance of the mSOFA in four prehospital ACVD groups (acute heart failure, ischemic heart disease, arrhythmia, and syncope).

## 2. Materials and Methods

### 2.1. Design and Study Setting

A prospective, observational, multi-center, ambulance-based, EMS-delivery, observational, controlled study was conducted in adults with suspected ACVD transferred by ambulance to an Emergency Department (ED) between 1 October 2019 and 30 November 2021.

This study was undertaken in three Spanish provinces (Salamanca, Segovia, Valladolid), covering a population of 995,137 residents. This study involved six advance life support (ALS) units, thirty-eight basic life support (BLS) units and four hospitals, all with an Acute Cardiac Care Unit (UCCA) and two with a 24/7 cardiac intervention room; therefore, in case of an unexpected requirement for emergency transfer to the hemodynamics unit, priority was given to the emergency relocation to one of these specialized centers. All facilities were managed by the Public Health System (SACYL).

Citizens request emergency medical support by calling the 1-1-2 phone number and an operator collects the geolocation and affiliation data. Subsequently, a coordinating physician conducts a brief guided consultation and assigns the most appropriate assistance option. The BLS teams are made up of two Emergency Medical Technicians (EMT), and the ALS teams include two EMT, an Emergency Registered Nurse (ERN) and a physician. These teams provide basic or advanced life support based on pre-established protocols and clinical practice guidelines, either on-scene or en route.

### 2.2. Participants

Recruitment was carried out back-to-back, considering adult patients (>18 years old) with a prehospital diagnosis of ACVD who were transferred to an ED by ambulance. Without exception, all patients included in this study were evaluated on-scene by an ALS and, following evaluation, the ALS physician either decided on no transfer (discharge on site), or alternatively that the case required emergency transfer to an ED, either by a BLS or an ALS. Minors, end-stage patients (documented by a specialist report), non-recovered cardiorespiratory arrest, pregnant women (at any period of gestation), cases where it was not possible to obtain prehospital analysis and patients without informed consent were excluded.

### 2.3. Outcomes

The primary outcome was mortality at 2 and 90 days (all-cause, and in- and out-hospital). As a secondary outcome, an additional analysis by syndromic groups was performed; patients were categorized according to the most relevant signs and symptoms of ACVD manifested during prehospital care. The categories used were: acute heart failure (AHF), ischemic heart disease (IHD), arrhythmia and syncope.

### 2.4. Data Sources and Predictors

During on-site evaluation, an ERN collected epidemiological variables (sex and age) and baseline vital signs (respiratory rate, oxygen saturation, blood pressure, heart rate, temperature, and level of consciousness), and also performed an electrocardiogram and prehospital blood analysis (creatinine, lactate). Neurological status was systematically monitored using the Glasgow Coma Scale (GCS). Respiratory rate was obtained by listening to the ventilatory cycles for 30 s or, in case of doubt or irregular rhythm, for 1 min. Delirium, inability to respond to commands and a GCS under 15 points were classified as altered mental status, a categorization matching AVPU (Alertness, Verbal, Pain, Unresponsive). Oxygen saturation, blood pressure, heart rate and electrocardiograms were obtained using a LifePAK^®^ 15 monitor-defibrillator (Physio-Control, Inc., Redmond, WA, USA), and the temperature was obtained using a ThermoScan^®^ PRO 6000 (Welch Allyn, Inc., Skaneateles Falls, NY, USA). POCT epoc^®^ (Siemens Healthcare GmbH, Erlangen Germany) was utilized to provide creatinine and lactate levels. In addition, on the basis of the signs and symptoms guide, the physician categorized the prehospital ACVD group into: AHF, IHD, arrhythmia and syncope.

In a second step, during a 90-day follow-up, an associate investigator from each hospital collected mortality data (2- and 90-day) and the 17 comorbidities’ categories required to calculate the Age-Adjusted Charlson comorbidity index (ACCI) (i.e., myocardial infarction, congestive heart failure, peripheral vascular disease, stroke or transient ischemic attack, dementia, chronic obstructive pulmonary disease, connective tissue disease, peptic ulcer disease, mild liver disease, uncomplicated diabetes mellitus, hemiplegia, moderate-to-severe chronic kidney disease, diabetes mellitus with end-organ damage, localized solid tumor, leukemia, lymphoma, moderate-to-severe liver disease, metastatic solid tumor, acquired immunodeficiency syndrome, unplanned ICU-admission, vascular interventional procedures, fibrinolysis, or emergency surgery).

Finally, based on the prospectively gathered data, the analyzed scores were calculated retrospectively; see the supplementary data p3 for details on how scores were calculated. The EMS-providers had no knowledge of the scores analyzed, and no score was employed to inform bedside decision-making during this study.

### 2.5. Statistical Analysis

The collected data were explored for normality using the Shapiro–Wilk test. The descriptive results and the association between predictors and the outcome were assessed using the Mann–Whitney U test or the chi-squared test, as appropriate, and the effect size was provided as the standardized mean difference. Absolute values and percentages were used for categorical variables, and medians and interquartile ranges (IQR) were used for continuous variables because they did not follow a normal distribution. Sample size calculations can be found in the supplementary data p4.

The evaluation of the mSOFA and the other scores required a first step of fitting a logistic regression in which the score (as a continuous variable) was the predictive variable and the 2-day mortality or 90-day mortality was the outcome. The process of determining the discriminative power and the calibration is described in the supplementary data p4. Taking the whole cohort into consideration, we plotted the observed distribution of the outcomes and a curve of the predicted probability of the outcome according to the scores, including the confidence interval.

To assess the reliability of the mSOFA and to compare it against other well-established scores, all the scores were evaluated in three different ways: with their discriminative power (assessed by the area under the receiver operating characteristic [(OC) curve (AUC)); their calibration (observed vs. predicted outcome agreement); and with the decision curve analysis (DCA, clinical utility) (supplementary data p4). 

Finally, the role of different confusion variables in the predictive power of the mSOFA was assessed by determining the AUC for each subset of patients, i.e., for sex, splitting the sample into males and females; for age, splitting the sample into the following age ranges: “18–49”,”50–60”,”60–75”,”75–85” and ”over 85”; and for pathology, splitting the sample into “AHF“, “IHD”, “arrhythmia” and “syncope”.

All statistical analyses were performed in R, version 4.0.3 (supplementary data p4).

### 2.6. Ethic Statements

This study was approved by the Health Research Ethics Board (ref. PI041-19, and PI217-20), and registered in the World Health Organization’s International Clinical Trials Registry Platform and is available online (doi.org/10.1186/ISRCTN48326533 (accessed on 16 February 2023) and doi.org/10.1186/ISRCTN49321933). This study is reported in line with the Strengthening the Reporting of Observational Studies in Epidemiology (STROBE) statement (supplementary data p17). All participants in this study have read and signed the informed consent.

## 3. Results

### 3.1. Baseline Characteristics

**Figure 1:**
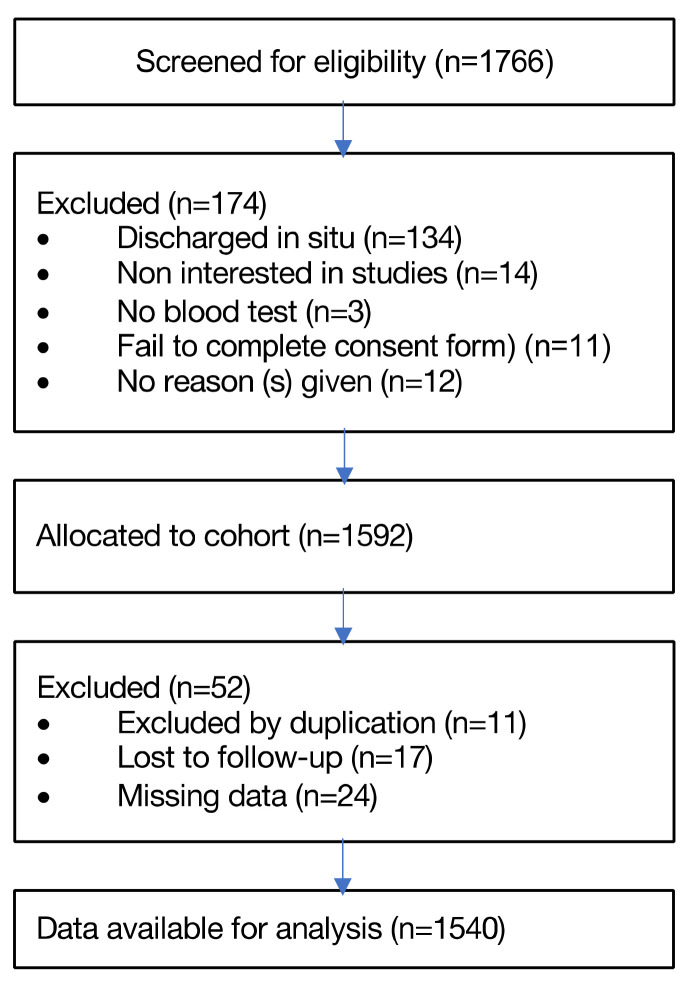
Flowchart.

A total of 1540 patients met the inclusion criteria. Figure 1 shows a flowchart the process used to select patients. 

The median age was 74 years old (IQR 62–83) and 640 (41.6%) patients were female. The 2-day mortality rate was 5.3%, and the 90-day mortality rate was 12.7%. The clinical and epidemiological characteristics of the patients and the differences between survivors and non-survivors by 2- and 90-day mortality are displayed in Table 1 and Table S1a,b (Supplementary Materials). Statistically significant differences were found between survivors and non-survivors for all variables except temperature at both 2 and 90 days. All variables from calculated scores showed significant differences.

### 3.2. mSOFA Performance vs. Early Warning Scores

**Figure 2:**
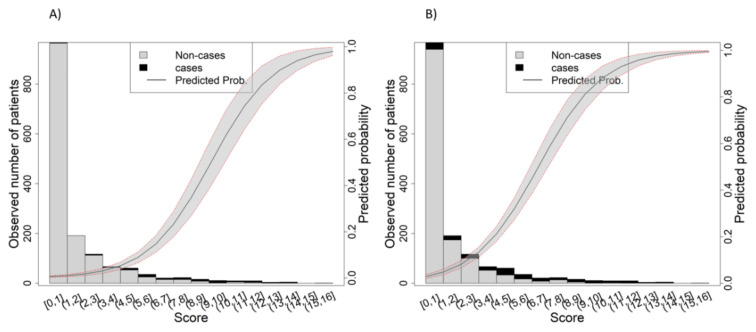
The mSOFA scores versus real and predicted probability of death. (**A**) mSOFA at 2-day mortality. (**B**) mSOFA at 90-day mortality. The grey area of the trend line corresponds to 95% confidence interval of the predicted probability of death (trend line). The bars correspond to the number of patients in the training cohort who were alive (grey) or dead (black). mSOFA: modified Sequential Organ Failure Assessment.

Taking 2-day mortality into consideration, the mSOFA presented a well-defined sigmoid-predicted probability curve when representing the observed vs. the score values, so higher values of the mSOFA reached a 100% probability of death (Figure 2A). For 90-day mortality (Figure 2B), the main difference is that the inflexion point occurs at lower mSOFA levels as compared to the 2-day mortality graph. Results of the representation of the observed vs. the score values are shown in Figure S1.

**Figure 3:**
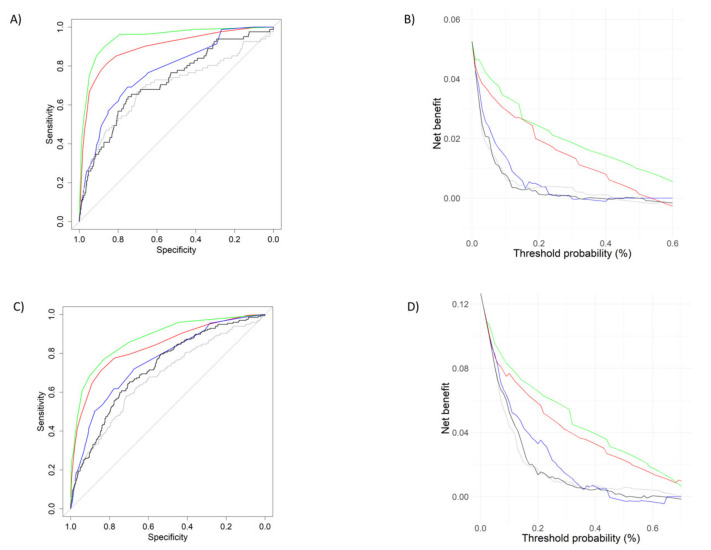
(**A**,**C**) Receiver operational curve (ROC) for mSOFA (green line), NEWS2 (red line), CART (blue line), TIMI (black line) and MSI (grey line) within 2- and 90-day mortality. (**B**,**D**) Decision curve of the mSOFA (green line), NEWS2 (red line), CART (blue line), TIMI (black line) and MSI (grey line) within 2- and 90-day mortality. The threshold probability for the mortality is shown on the *x*-axis and the *y*-axis indicates the net benefit. Abbreviations: mSOFA: modified Sequential Organ Failure Assessment; NEWS2: National Early Warning Score 2; TIMI: TIMI risk index; MSI: modified shock index, CART: Cardiac Arrest Risk Triage.

According to predictive validity, the mSOFA showed the highest AUC of all the evaluated scores for both 2- and 90-day mortality, AUC = 0.943 (0.917–0.968) and AUC = 0.874 (0.847–0.902), respectively. Further details derived from AUC can be found in Table S2. Direct comparison of AUCs confirmed the mSOFA possessed the highest AUC, showing statistically significant differences between mSOFA’s AUC and the other scores’ AUC (Table 2). This can also be observed in the graphical representation of AUCs (Figure 3A,C). Similarly, the DCA results confirmed the superiority of the mSOFA as compared to the other scores (Figure 3B,D), with the mSOFA having a higher net benefit throughout all the thresholds when compared to the other scores, for both outcomes.

The calibration results showed that the mSOFA performed best for both 2- and 90-day mortality. This can be observed by considering the Brier score, in which the mSOFA reached the lowest value (Figure S2a,b). Interestingly, the fitted calibration curves, whether logistic or nonparametric (LOWESS), exemplified the proper fit of the mSOFA, and as was the case with the previous analysis, only NEWS2 presented a similar fitment.

### 3.3. mSOFA according to Different Confusion Factors

Finally, in order to rule out the possible influence of confusion factors such as age, sex and pathology, the discrimination capacity was assessed using the AUC (Figure S3a–d). When considering the 2-day mortality outcome, no differences were found in the AUC, all of them surpassing 0.9 except for the heart failure AUC = 0.891 (0.834–0.948). Results for the 90-day outcome were more variable; no huge differences were found for sex or age, except for the >85 years groups which reached AUC = 0.775 (0.703–0.847). For the pathologies, arrhythmia was the group that presented the lowest AUC, 0.771 (0.618–0.923).

## 4. Discussion

This multicenter, observational, prospective study is the first to evaluate the ability of the mSOFA to predict 2-day mortality (all-cause) in patients treated with ACVD in prehospital care. Compared to the rest of the scores analyzed, the predictive capacity displayed by the mSOFA was significantly superior, showing an excellent capacity to predict 2-day mortality (AUC = 0.94), and a satisfactory capacity to recognize patients with a high risk of 90-day mortality (AUC = 0.87).

The mSOFA was originally developed to be used by EMS as a quicker and simpler alternative to SOFA, providing a similar correlation with ICU admission and mortality [9,10]. Currently other versions of the mSOFA, which are different from the one used in this investigation, are available. These are designed for ambulatory use but include platelets and bilirubin [11,12], which are analytical parameters technically impossible to calculate on-scene or en route. However, this investigation was performed with a customized version of the mSOFA which was specially adapted for prehospital care. This version is shown to accurately estimate mortality [9] and is made up of SaFi instead of PaFi (arterial oxygen pressure/inspired oxygen fraction ratio); mean arterial pressure (exclusively, not considering vasoactive drug use); GCS and creatinine, and includes adding lactate to assess metabolic-oxidative function. To our knowledge, the mSOFA has not yet been exclusively applied in prehospital care in ACVD patients, despite higher prevalence and mortality in these patients.

The study performed to develop the mSOFA, described by Martín-Rodríguez et al., is the only one that applied the mSOFA in prehospital care, yielding an AUC for target 2-day mortality of 0.94—the same as our results [9]. In this sense, our current evaluation focused on patients with ACVD, whereas the original investigation referred to any acute disease. Our results are in agreement with previous studies, such as that of Ebrahimnian A. et al. [13], which evaluated the ability of another version of the mSOFA to predict all-cause mortality in ED in patients transferred for non-traumatic causes, obtaining an AUC of 0.923; or that of Grissom C. et al. who obtained an AUC of 0.84 for all-cause mortality at 30 days, although the AUC for predicting mortality at 3 days was lower (0.79) [12].

The performance of the mSOFA was adequate in all age ranges, sex and pathological groups, although when discarding the influence of possible confounding factors we found that its performance at 90 days decreased in patients older than 85 years. This is somewhat expected, since these patients, who have a shorter life expectancy, may have died during follow-up due to other intercurrent processes not assessable by the mSOFA. In addition, the mSOFA’s performance was also lower in patients seen for causes belonging to the arrhythmia diagnostic group, which includes pathologies that a priori may be milder and who may have died from other causes.

The mSOFA also proved to be superior to all other EWS studied, having better AUC, calibration curves and DCA at both 2 and 90 days. However, all of them also demonstrated acceptable predictive ability. The comparison of our data with previous evidence is difficult because although these EWS are now part of routine clinical practice in many EMS, the vast majority have been developed and extensively studied in hospital settings [2,14] so there is little evidence relating to their use in prehospital settings and not all EWS are validated for this use [15]. Moreover, many of the EWS have been developed and implemented for the assessment of a specific pathology or group of pathologies (TIMI for STEMI and non-STEMI patients [16,17], or CART for predicting cardiac arrest [18]), so their application to all causes of ACVD has never been studied.

The meta-analysis by Guan G. et al. [19] was conducted in Australia and studied the ability of several EWS in both in-hospital and prehospital settings to predict a composite outcome of ICU-admission and mortality at 3 and 30 days in patients with all-cause conditions. That study concluded there was inferior performance in the outpatient environment, with higher cutoff points and worse predictive ability. However, we tested certain EWS covered in Guan G. et al., e.g., NEWS2, and obtained significantly improved prehospital data at 2 and 90 days (AUC of 0.84 and 0.75 at 3 and 30 days, vs. 0.89 and 0.83 at 2 and 90 days in our study). This could be explained by the fact that the majority of EMS systems are provided by EMT or paramedics [19,20], and, after initial stabilization, patients, including those with milder symptoms and, a priori, with a better prognosis, were transferred to an ED,. Nevertheless, in Spain, the standard ALS is staffed by a physician, an ERN and two EMT, expanding bedside decision-making capacity, with the physician deciding on discharge in situ or transfer to the ED (in BLS or ALS), resulting in a significant percentage of cases being transferred to the ED with more serious discomfort. Original NEWS and upgraded NEWS2 (2017) are two of the most well-studied and widely-used EWS, both in EDs and EMS, and have proven to be a valuable tool for out-of-hospital risk assessment, being standardized in countries such as the United Kingdom and endorsed by the Royal College of Physicians [21]. In our study, NEWS2 emerged as the score with the best prognostic ability after the mSOFA for all ACVD, age and sex groups. A recent systematic review by Burgos-Esteban et al. [22] analyzed the performance of several EWS in the out-of-hospital scenario, obtaining similar results for NEWS with data in line with comparable reports and with an AUC above 0.85 in detecting short-term clinical impairment risk (1-day mortality) [23,24].

### Limitations

This study has several limitations. Firstly, the data collectors were not blinded so it was decided that, to minimize bias, samples would be collected on a criterion of opportunity. Secondly, there is currently no gold-standard EWS with which to compare the mSOFA, so it was decided to use NEWS, MSI, TIMI and CART as these scores are widely used in EDs and EMS, although this is still a partial and subjective decision. Thirdly, the prehospital diagnosis of ACVD may not coincide with that made in hospital, as in our setting there is usually less time, fewer resources and less information available to assess patients. It was therefore decided to categorize patients into four diagnostic groups (IHD, AHF, syncope, and arrhythmia). Finally, although the sample was sufficient to obtain preliminary results, it would be interesting to perform studies in different EMS with larger samples, both to generalize the results and to check the concordance between pre- and in-hospital diagnoses.

## 5. Conclusions

EWS are becoming increasingly popular tools used by EMS to target high-risk-mortality patients in prehospital care. The mSOFA is a quick and easy-to-use EWS with an excellent ability to predict mortality at both 2 and 90 days in patients treated for ACVD, outperforming the other EWS evaluated in this study.

Although further studies with larger samples would be necessary to validate this score and determine mSOFA’s usefulness in different subgroups of cardiovascular pathologies, implementation of the mSOFA in EWS could help professionals to optimize the assessment and management of patients suffering from time-dependent ACVD.

## Figures and Tables

**Table 1:** Demographic and clinical baseline variables for 2-day mortality.

		2-Day Mortality		
Variable	Total	Survivors	Non-Survivors	*p* Value ^2^
No. (%) with data ^1^	1540	1459 (94.7)	81 (5.3)	N.A.
Epidemiological variables				
Sex, female	640 (41.6)	610 (41.8)	30 (37)	0.369
Age, year	74 (62–83)	74 (53–95)	79 (65–86.5)	0.139
On-scene vital signs				
Respiratory rate, bpm	17 (14–21)	17 (14–20)	26 (14.5–35)	<0.001
Oxygen saturation, %	96 (94–98)	97 (94–98)	86 (74.5–94)	<0.001
FiO2, %	0.21 (0.21–0.21)	0.21 (0.21–0.21)	0.21 (0.21–0.28)	<0.001
SaFi	457 (442–466)	457 (447–466)	366 (255–423)	<0.001
SBP, mmHg	134 (122–155)	136 (115–156)	100 (76–141)	<0.001
DBP, mmHg	78 (64–91)	78 (65–91)	60 (44–87)	<0.001
MBP, mmHg	97 (82–111)	97 (83–112)	73 (57–104)	<0.001
Heart rate, beats/min	79 (64–91)	79 (64–100)	97 (68–132)	<0.001
Temperature, °C	36 (35.8–36.5)	36 (35.8–36.5)	36.1 (35.15–36.5)	0.355
GCS, points	15 (15–15)	15 (15–15)	12 (5–15)	<0.001
Creatinine, mg/dL	0.98 (0.81–1.31)	0.97 (0.79–1.24)	1.99 (1.145–2.80)	<0.001
Lactate, mmol/L	1.86 (1.15–2.94)	1.83 (1.14–2.83)	7.21 (4.91–12.05)	<0.001
Prehospital syndromic				
Ischemic heart disease	637 (41.4)	608 (41.7)	29 (35.8)	<0.001
Acute heart failure	281 (18.2)	245 (16.8)	36 (44.4)	<0.001
Arrhythmia	187 (12.1)	182 (12.5)	5 (6.2)	<0.001
Syncope	435 (28.2)	424 (29.1)	11 (13.6)	<0.001
Hospital outcomes				
ACCI, points	5 (3–7)	5 (3–7)	7 (5–9)	<0.001
Inpatient	830 (53.9)	751 (51.5)	79 (97.5%)	<0.001
Fibrinolysis	35 (2.3)	23 (1.6)	12 (14.8)	<0.001
PIVS	361 (23.4)	339 (23.1)	22 (27.1)	0.417
Emergent surgery	38 (2.5)	27 (2.5)	1 (1.2)	0.463
ACCU-admission	370 (24.0)	328 (22.5)	42 (51.9)	<0.001
Scores calculation, points				
mSOFA	1 (0–2)	1 (0–2)	8 (5–10)	<0.001
TIMI risk index	8 (6–12)	8 (6–11)	13 (8–19)	<0.001
Modified shock index	0.82 (0.65–1.08)	0.81 (0.65–1.05)	1.21 (0.77–1.74)	<0.001
Cardiac Arrest Risk Triage	9 (4–17)	9 (4–16)	25 (12–35)	<0.001
NEWS2	3 (1–6)	3 (1–6)	12 (9–15)	<0.001

^1^ Values expressed as total number (percentage) and medians (25 percentile-75 percentile), as appropriate. ^2^ The Mann–Whitney U test or Chi-squared test was used as appropriate. Abbreviations: NA: Not applicable; FiO_2_: Fraction of inspired oxygen; SaFi: Oxygen saturation/Fraction of inspired oxygen ratio; SBP: systolic blood pressure; DBP: diastolic blood pressure; MBP: mean blood pressure; GCS: Glasgow coma scale; ACCI: Age-Adjusted Charlson comorbidity index; PIVS: Percutaneous Interventional Vascular Surgery; ACCU: Acute Cardiac Care Unit; mSOFA: modified Sequential Organ Failure Assessment; NEWS2: National Early Warning Score2.

**Table 2:** AUC comparison between different scores for the three different outcomes. (**a**) 2-day mortality, (**b**) 90-day mortality.

(a)	mSOFA	NEWS2	CART	MSI	TIMI
mSOFA	0.94 (0.91–0.96)				
NEWS2	0.011	0.89 (0.85–0.94)			
CART	<0.001	<0.001	0.77 (0.72–0.82)		
MSI	<0.001	<0.001	0.032	0.69 (0.62–0.76)	
TIMI	<0.001	<0.001	0.095	0.047	0.71 (0.65–0.78)
**(b)**	**mSOFA**	**NEWS2**	**CART**	**MSI**	**TIMI**
mSOFA	0.87 (0.84–0.90)				
NEWS2	0.011	0.83 (0.79–0.86)			
CART	<0.001	<0.001	0.75 (0.72–0.79)		
MSI	<0.001	<0.001	<0.001	0.67 (0.63–0.71)	
TIMI	<0.001	<0.001	0.061	<0.001	0.71 (0.68–0.75)

The table shows the *p*-values (Delong’s test) of each comparison. The diagonal (bold values) shows the AUC and 95% confidence interval.

## Data Availability

The data of the study are available to other researchers, upon reasonable request to the corresponding author.

## Supplementary Materials

The following supporting information can be downloaded at https://www.mdpi.com/article/10.3390/jcdd10020088/s1: Supplementary Methods (Scores Calculation, Sample Size, Software, Scores Evaluation); Table S1: Basal electrocardiographic rhythm and cardioversion on-scene; Table S2: Further parameters of ROC curve analysis of mSOFA for different outcomes; Figure S1: Probability of the outcome based on the score value; Figure S2: Calibration of each score; Figure S3: ROC curve analysis of mSOFA. References [25,26,27,28,29,30] are cited in the Supplementary Materials.
