# Supplementary material for: Modified Sequential Organ Failure Assessment Score vs. Early Warning Scores in Prehospital Care to Predict Major Adverse Cardiac Events in Acute Cardiovascular Disease
Source: J Cardiovasc Dev Dis. 2023 Feb 17;10(2):88. doi: 10.3390/jcdd10020088 (PMC9966856; doi:10.3390/jcdd10020088)
Supplement: Supplementary file 1 [file jcdd-10-00088-s001.zip › jcdd-2225049-supplementary.pdf]

Supplementary Data

Supplementary Data

Table of contents

Supplementary methods…………………………………………………………………3

Scores calculation…………………………...…………………………………….…3

Sample size…………………………………………………………………...4

Software………………………………………………………………….....4

Scores Evaluation…………………………………………………………4

Supplementary Results…………………………………………………………..5

- Supplementary Table S1a: Basal electrocardiographic rhythm on-scene.……..5

- Supplementary Table S1b. Basal cardioversion on-scene…………………..….5

- Supplementary figure S1: Probability of the outcome based on the score value…………………………………………………………………………...6

- Supplementary Table S2: Further parameters of ROC curve analysis of mSOFA for different outcomes…………………………………………..…...7

- Supplementary figure S2a: Calibration of each score 2-day mortality.………..8

- Supplementary figure S2b: Calibration of each score 90-day mortality.………9

- Supplementary figure S3a: ROC curve analysis of mSOFA for 2-day mortality according to age range and sex……………………………………………..….10

- Supplementary figure S3b: ROC curve analysis of mSOFA for 2-day mortality according to pathology………..……………………………………………..…11

- Supplementary figure S3c: ROC curve analysis of mSOFA for 90-day mortality according to age range and sex...……………………………………12

- Supplementary figure S3d: ROC curve analysis of mSOFA for 90-day mortality according to pathology……….……………………………………..…13

STROBE Statement—checklist of items that should be included in reports of observational studies………………………………………………………………14

References..18

Supplementary Methods

Scores Calculation.

mSOFA	Points 0	1	2	3	4
Respiratoriy, SaFI	>302	<302	<221	<142	<67
Cardiovascular, MAP (mmHG)	≥70	<70			
Renal, Creatinine (mg/dL)	<1.2	1.2-1.9	2.0-3.4	3.5-4.9	>5.0
Neurologic, GCS (points)	15	13-14	10-12	6-9	<6
Metabolic, Lactate (mmol/L)	<2	2.1-3	3.1-4	4.1-6	>6

CART score	Score	Points
RR, breaths/min	<21	0
	21-23	+8
	24-25	+12
	26-29	+15
	>29	+22
HR, beats/min	<110	0
	110-139	+4
	>139	+13
DBP, mmHg	>49	0
	40-49	+4
	35-39	+6
	<35	+13
Age, years	<55	0
	55-69	+4
	>69	+9

TIMI risk index: heart rate × [age/10]2/systolic blood pressure

Modified shock index: Heart rate/ mean blood pressure

NEWS2	Score						
	3	2	1	0	1	2	3
Respiration rate (per minute)	≤8		9-11	12-20		21-24	≥25
SpO2 Scale 1 (%)	≤91	92-93	94-95	≥96			
SpO2 Scale 2 (%)	≤83	84-85	86-87	88-92<br>≥93 on air	93-94 on oxygen	95-96 on oxygen	≥97 on oxygen
Air or oxygen?		Oxygen		Air			
Systolic blood pressure (mmHg)	≤90	91-100	101-110	111-219			≥120
Pulse (per minute)	≤40		41-50	51-90	91-110	111-130	≥131
Consciousness				Alert			CVPU
Temperature (ºC)	≤35.0		35.1-36.0	36.1-38.0	38.1-39.0	≥39.1	

Sample Size

Based on previous studies[2], the statistical power (from 1 to 100) of the present study for each individual score is equal to 100 based on the following considerations: (i) the sample used n = 1540, (ii) significant level of p = 0.001, (iii) an mSOFA difference between cases and non-cases of 75%.

Software

All calculations and analyses were performed by using our own codes, R packages and base functions in R, version 4.0.3 (http://www.R-project.org; the R Foundation for Statistical Computing, Vienna, Austria). In particular, the following packages were used: pROC (version 1.16.2)[3], for C-statistic calculations, rms (version 6.2-0)[4] for calibration metrics calculation.

Scores Evaluation

In particular, the mSOFA (and other scores) discrimination capacity was assessed by the area under the receiver operating characteristic (ROC) curve (AUC). The calibration was also performed by calculating the calibration curve, that is, plotting predicted vs observed probability of the outcome, and determining several metrics associated to calibration (explained below).

The discriminative power of mSOFA (and other scores) was assessed by ROC curve analysis and AUC, including the 95% confidence intervals (CI) and the p value of the hypothesis testing (H0: AUC=0.5). All 95% CI were obtained by bootstrapping (2000 iterations). Further parameters of the ROC were assessed: specificity (sp), sensitivity (sen), positive predictive value, negative predictive value, positive likelihood ratio, and negative likelihood ratio. The maximum potential effectiveness achieved by the scores, the Youden Index (in terms of sensitivity and specificity, serving also as a summary of the whole ROC curve) were also reported. AUCs were compared by the Delong's test.

The calibration[5] of the score, which represents the predicted vs observed probability of the outcome, should be as close to the diagonal as possible to rule out possible over- or under-estimation of the evaluated score (predicted probability) as compared to the ground truth (observed probability). The goodness of fit of the model against the observed probability (grey diagonal) was analyzed by using different types of adjustments: logistic (solid line) and nonparametric (dashed line). Additionally several statistics are calculated: Somers' D rank correlation (Dxy), ROC area (C), Nagelkerke-Cox-Snell-Maddala-Magee R-squared index (R2), Discrimination index D (D), Unreliability index (U), the quality index (Q), Brier score (average squared difference in p and y) (Brier), Intercept, Slope, maximum absolute difference in predicted and loess-calibrated probabilities (Emax), the average of the previous parameter (Eavg), the 0.9 of the previous parameter (E90), the Spiegelhalter Z-test for calibration accuracy (S:z), and its two-tailed P-value (S:p).

The decision curve analysis (DCA) allows to compare prediction models, it was performed by using the code from Vickers AJ et al.[6,7]. In particular, DCA deals with the clinical consequences of using new models as compared to those already used in clinical practice. Through the calculations of net benefit (of using a particular model compared to another) weighted by the odds of the outcome at a given threshold probability allows to plot benefit (y-axis) against preference (x-axis).

Supplementary Results

Supplementary Table S1a. Basal electrocardiographic rhythm on-scene.

		2-day mortality	
Rhythm[1]	**Total**	**Survivors**	**Non-survivors**
Sinus	697 (45.9)	685 (47.7)	12 (14.8)
Sinus arrhythmia	0 (0)	0 (0)	0 (0)
Atrial fibrillation	385 (25.4)	357 (24.9)	28 (34.6)
Atrial flutter	14 (0.9)	13 (0.9)	1 (1.2)
Atrial tachycardia	137 (9)	123 (8.6)	14 (17.3)
Supraventricular tachycardia	32 (2.1)	32 (2.2)	0 (0)
Ventricular tachycardia	22 (1.5)	16 (1.1)	6 (7.4)
Sinus bradycardia	93 (6.1)	88 (6.1)	5 (6.2)
First degree AV block	26 (1.7)	25 (1.7)	1 (1.2)
Type I 2 degree AV block	4 (0.3)	4 (0.3)	0 (0)
Type II 2 degree AV block	10 (0.7)	10 (0.7)	0 (0)
Third degree AV block	34 (2.2)	30 (2.1)	4 (4.9)
Pacemaker	42 (2.8)	40 (2.8)	2 (2.5)
AV junctional	4 (0.3)	2 (0.1)	2 (2.5)
Idioventricular	3 (0.2)	1 (0.1)	2 (2.5)
Right bundle-branch block	0 (0)	0 (0)	0 (0)
Left bundle-branch block	3 (0.2)	0 (0)	3 (0.2)
Ventricular extrasystoles	0 (0)	0 (0)	0 (0)
Asystole	4 (0.3)	0 (0)	4 (0.3)
Ventricular fibrillation	7 (0.5)	3 (0.2)	12 (14.8)

[1] Values expressed as total number (percentage).
Abbreviations: AV: Atrioventricular.

Supplementary Table S1b. Basal cardioversion on-scene.

		2-day mortality	
	**Total**	**Survivors**	**Non-survivors**
Cardioversion[1]	35 (2.27)	27 (1.87)	8 (9.87)

[1] Values expressed as total number (percentage).
Abbreviations: AV: Atrioventricular.

Supplementary Figure S1: Probability of the outcome based on the scores value. a) NEWS2 at 2-day mortality, b) NEWS2 at 90-day mortality, c) CART at 2-day mortality, d) CART at 90-day mortality, e) Shock index at 2-day mortality, f) Shock index at 90-day mortality, g) TIMI at 2-day mortality, h) TIMI at 90-day mortality. The solid line shows the predicted probability of the outcome; grey shadowed area shows the 95% confidence interval.

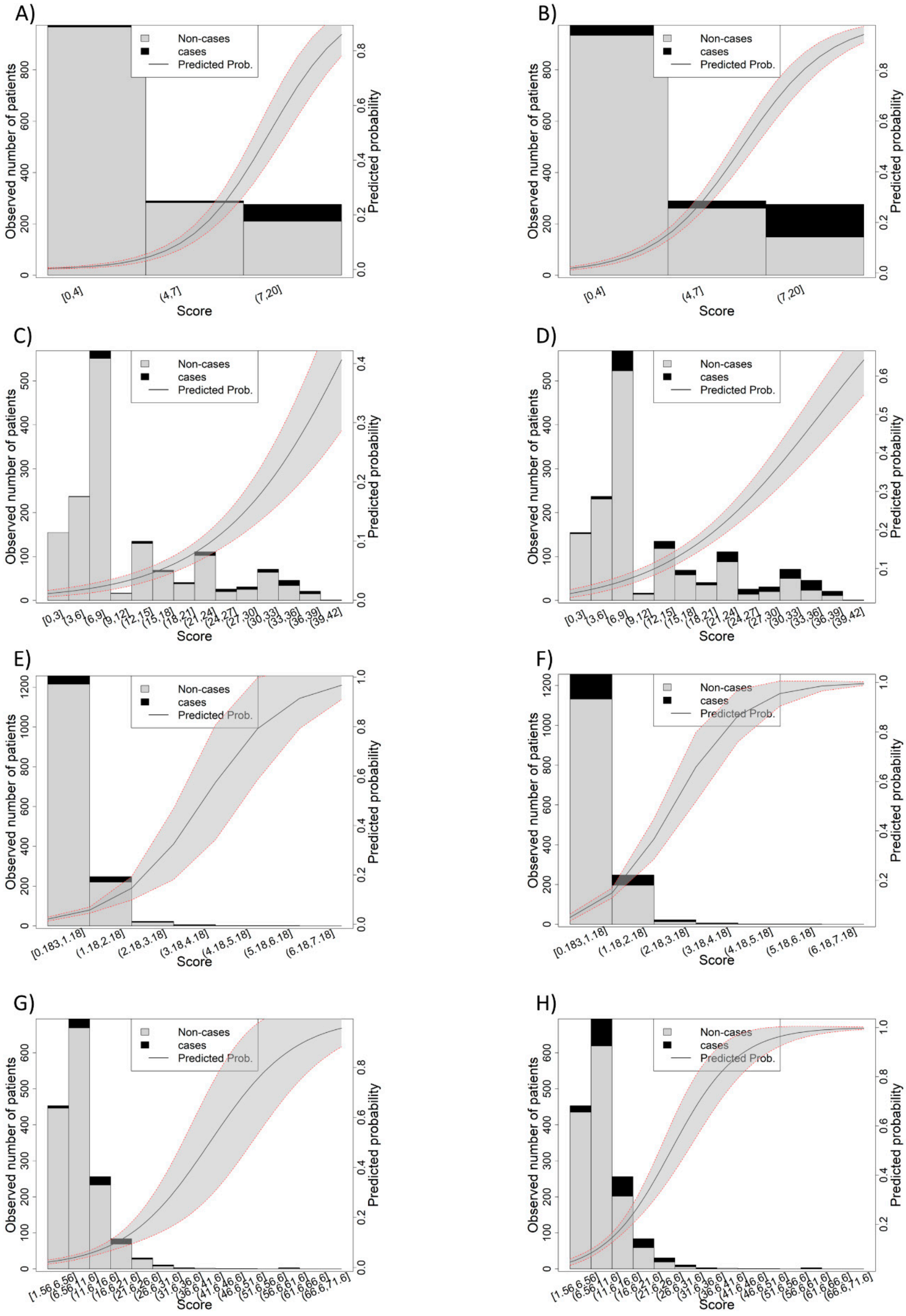

Supplementary Table S2: Further parameters of ROC curve analysis of all the scores for different outcomes. a) 2-day mortality and b) 90-day mortality. Note: the columns including "youden", refer to the maximum potential effectiveness achieved by the scores, i.e., the Youden Index, in terms of sensitivity and specificity, and the threshold at which these values are achieved. *Abbreviations*: sp: specificity; sen, sensitivity; ppv: positive predictive value; npv: negative predictive value; plr: positive likelihood ratio; npr: negative likelihood ratio. Values between parenthesis refer to 95% confidence interval.

a)

	Sp	sen	ppv	npv	plr	nlr	threshold (youden)	sp (youden)	sen (youden)
mSOFA	85.26 (71.41-99.12)	52.42 (32.7-72.13)	38.86 (25.16-52.56)	97.26 (96.22-98.293)	17.74 (9.5-25.95)	6.36 (-6.04-18.77)	3.5	86.84	90.12
NEWS2	76.26 (61.83-90.68)	59.37 (42.94-75.80)	29.43 (21.47-37.38)	87.58 (96.74-98.42)	9.16 (5.91-12.40)	5.19 (-4.69-15.08)	7.5	85.60	81.48
CART	71.40 (62.07-80.73)	54.34 (44.40-64.29)	17.34 (14.69-20.00)	97.19 (96.69-97.69)	4.03 (3.29-4.78)	2.73 (-1.72-7.19)	16.5	75.11	69.13
Shock index	85.10 (55.96-114.25)	21.91 (-7.06-51.43)	46.72 (16.83-76.61)	95.19 (94.56-95.82)	31.39 (-4.27-67.06)	13.29 (-15.9-42.58)	0.99	70.32	66.66
TIMI	88.91 (83.01-94.81)	18.81 (11.98-25.64)	44.84 (37.69-52.00)	95.37 (95.15-95.59)	27.58 (19.01-36.07)	2.19 (-0.43-4.83)	11.18	74.91	64.19

b)

	Sp	sen	ppv	npv	plr	nlr	threshold (youden)	sp (youden)	sen (youden)
mSOFA	86.65 (72.92-100.38)	39.82 (21.82-57.82)	68.28 (53.64-82.92)	91.40 (89.39-93.42)	40.83 (19.41-62.24)	6.50 (-5.88-18.88)	2.5	82.81	77.04
NEWS2	77.84 (63.44-92.24)	49.34 (33.29-65.38)	51.72 (39.96-63.49)	92.44 (90.66-94.21)	13.47 (7.18-19.76)	5.30 (-4.57-15.18)	6.5	84.52	70.91
CART	72.90 (63.58-82.21)	49.48 (39.41-59.56)	31.46 (28.19-34.73)	92.18 (91.10-93.27)	3.39 (2.91-3.87)	2.80 (-1.65-7.25)	16.5	77.82	61.73
Shock index	85.35 (56.16-114.54)	19.45 (-9.52-48.42)	70.88 (40.64-101.13)	87.95 (87.05-88.86)	54.00 (12.60-95.40)	13.32 (-15.9-42.60)	0.98	71.20	58.16
TIMI	89.36 (83.52-95.19)	17.32 (10.51-24.14)	67.62 (60.50-74.74)	88.72 (88.15-89.29)	43.77 (33.55-53.98)	2.19 (-0.43-4.83)	10.49	69.71	65.30

Supplementary figure S2a: Calibration of each score 2-day mortality for a) mSOFA, b) NEWS2, c) CART, d) Shock index, e) TIMI.

A)

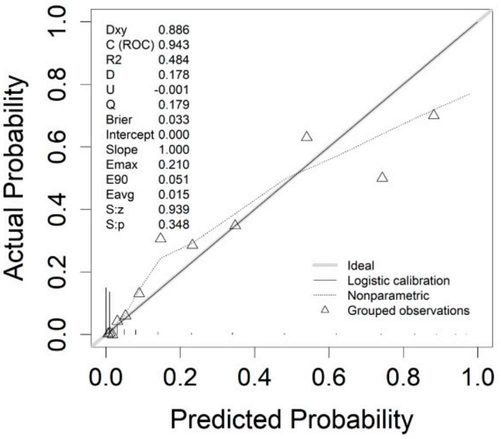

B)

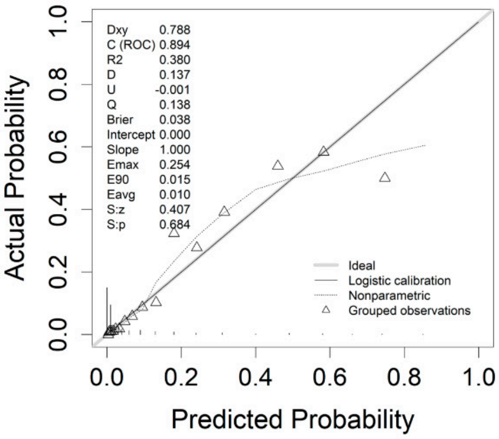

C)

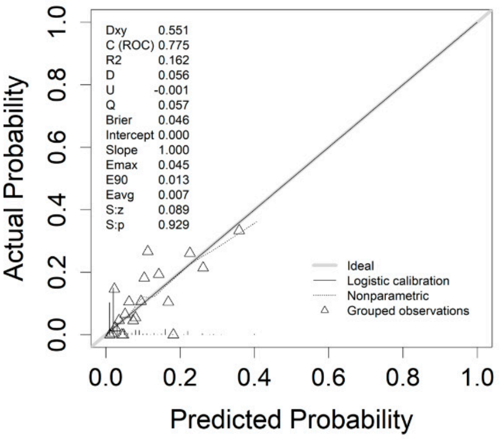

D)

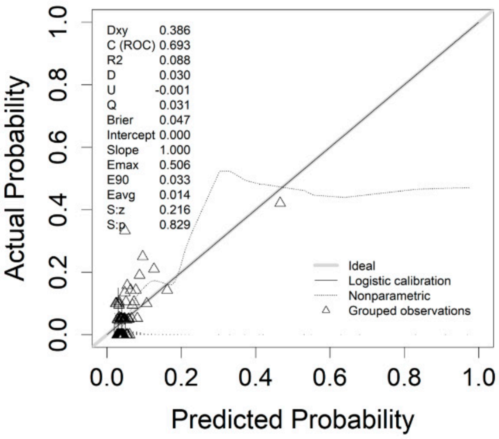

E)

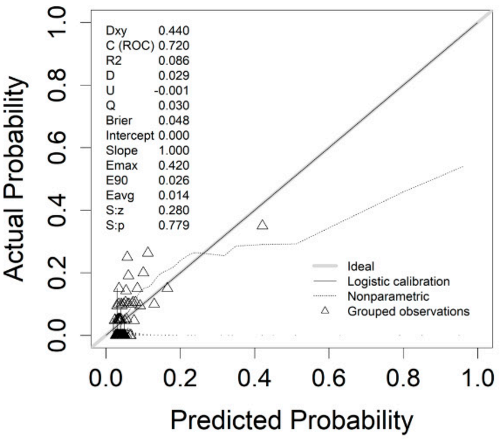

Supplementary figure S2b: Calibration of each score 90-day mortality for a) mSOFA, b) NEWS2, c) CART, d) Shock index, e) TIMI.

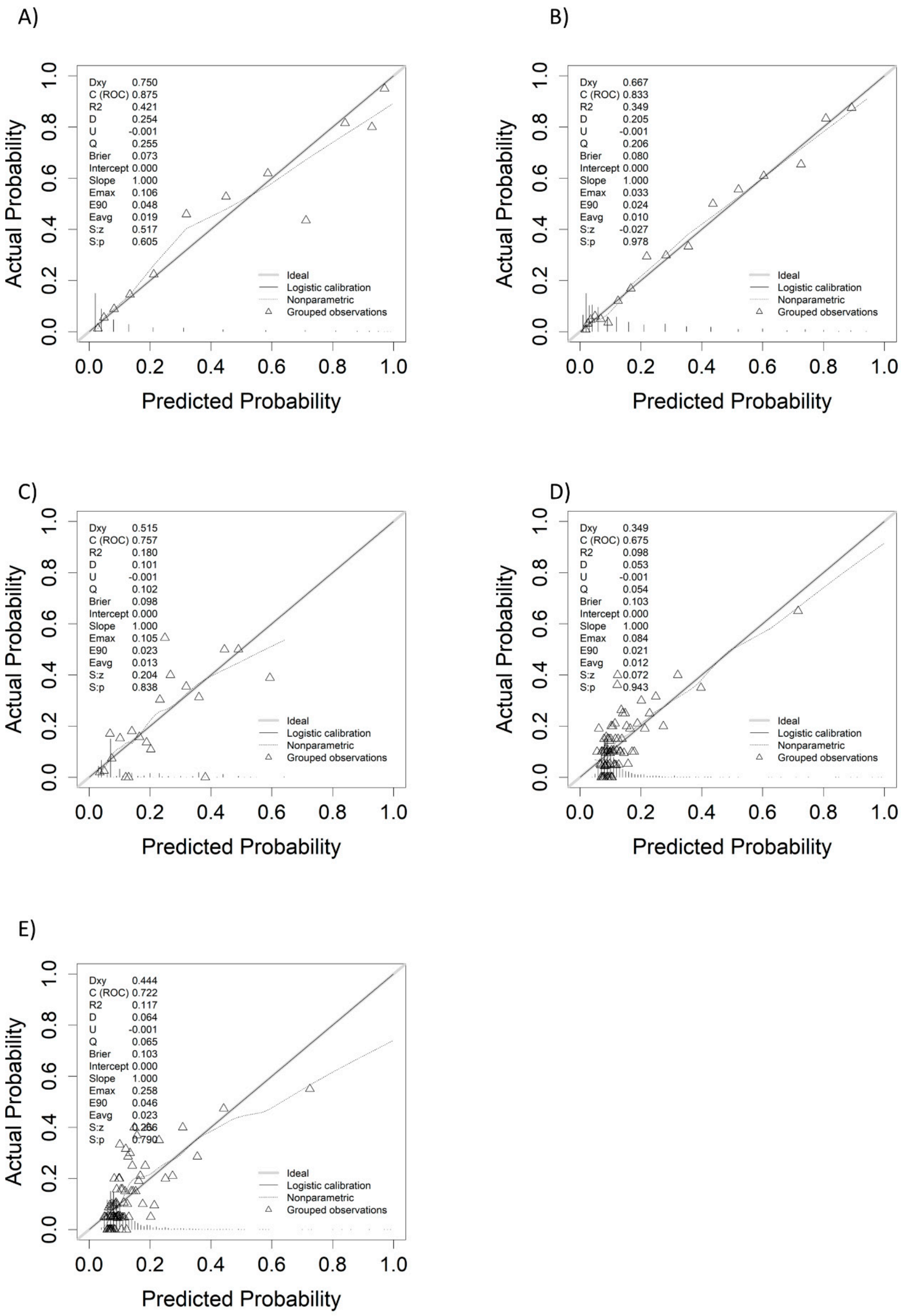

Supplementary figure S3a: ROC curve analysis of mSOFA for 2-day mortality according to age range and sex

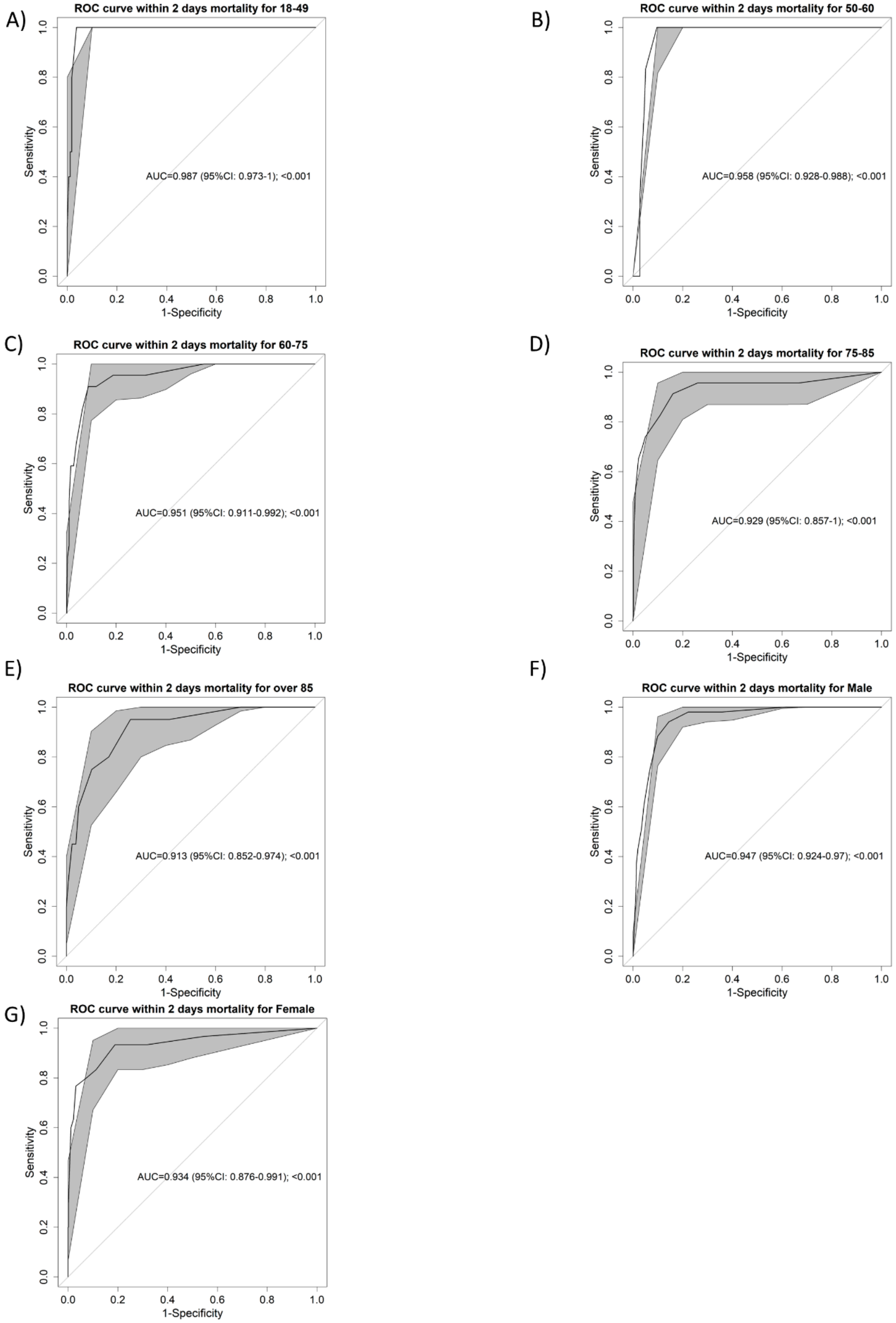

Supplementary figure S3b: ROC curve analysis of mSOFA for 2-day mortality according to pathology

A)

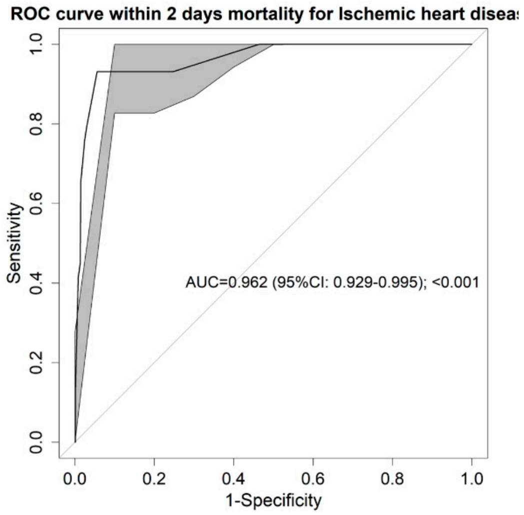

B)

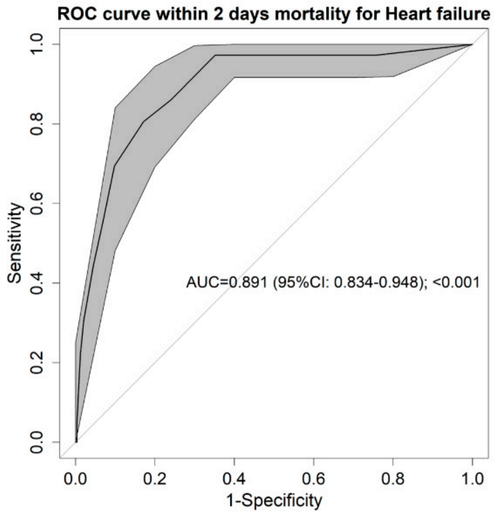

C)

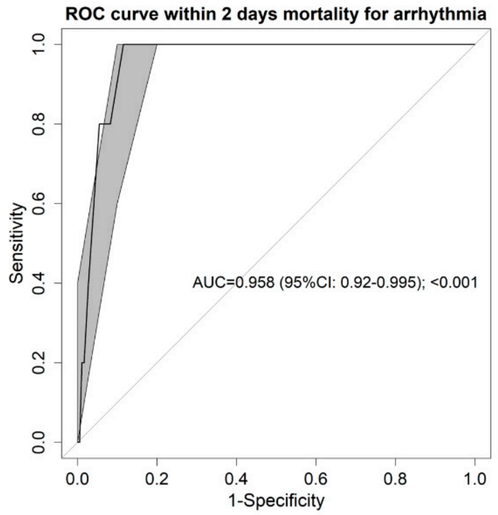

D)

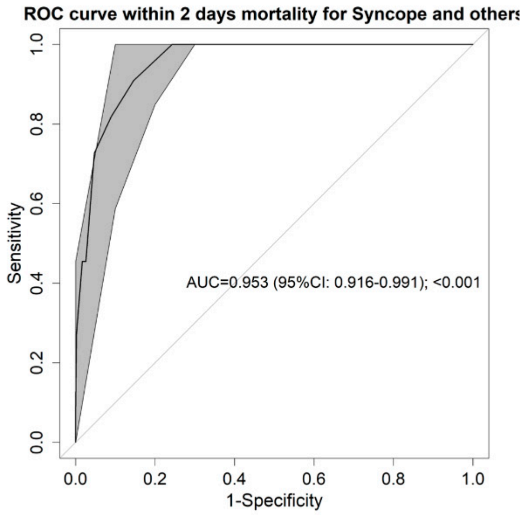

Supplementary figure S3c: ROC curve analysis of mSOFA for 90-day mortality according to age range and sex

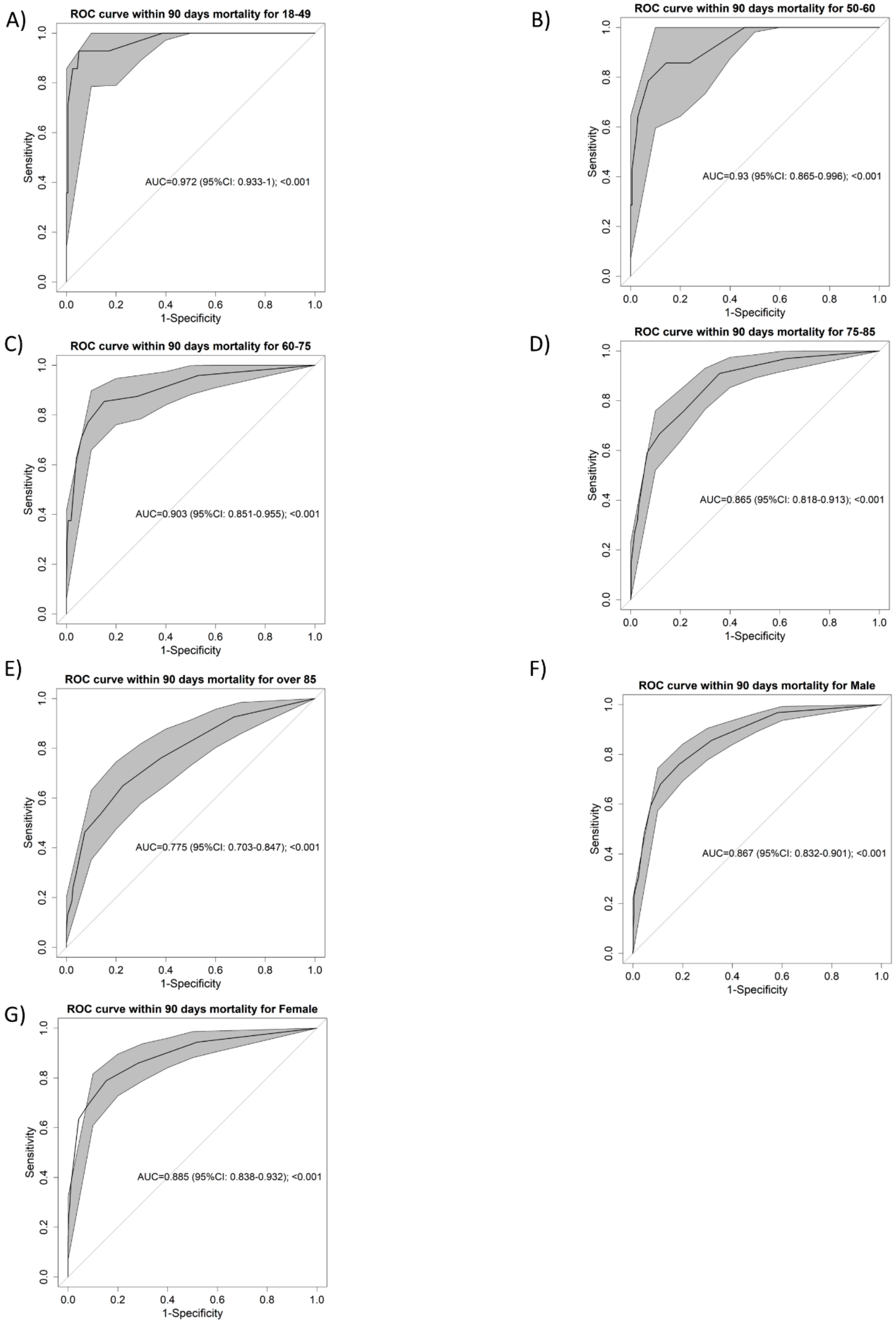

Supplementary figure S3d: ROC curve analysis of mSOFA for 90-day mortality according to pathology

A)

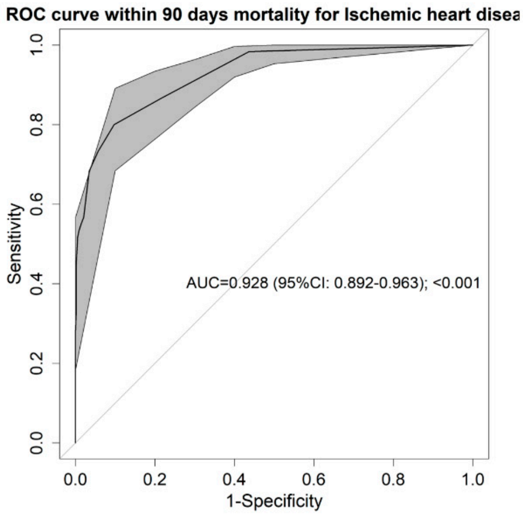

B)

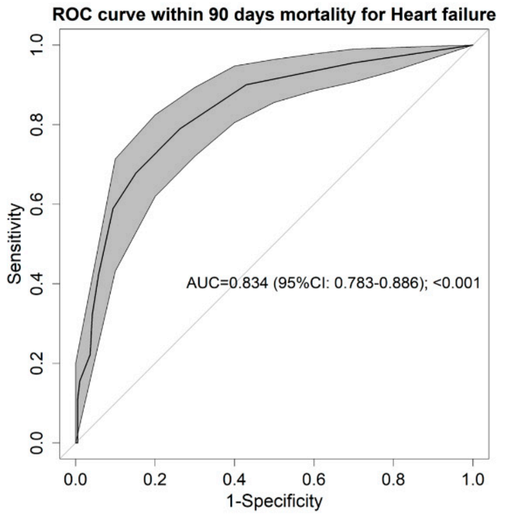

C)

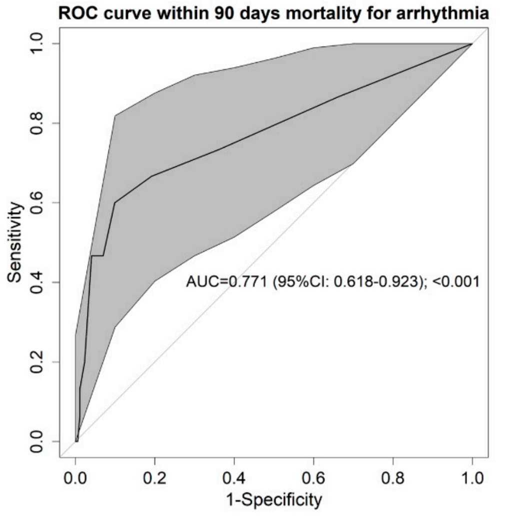

D)

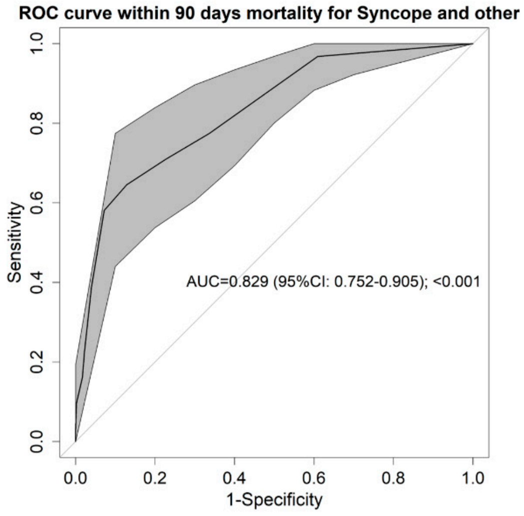

STROBE Statement—checklist of items that should be included in reports of observational studies

	Item No	Recommendation	Page No
**Title and abstract**	1	(*a*) Indicate the study's design with a commonly used term in the title or the abstract	1
		(*b*) Provide in the abstract an informative and balanced summary of what was done and what was found	1
**Introduction**			
Background/rationale	2	Explain the scientific background and rationale for the investigation being reported	2
Objectives	3	State specific objectives, including any prespecified hypotheses	2
**Methods**			
Study design	4	Present key elements of study design early in the paper	3-4
Setting	5	Describe the setting, locations, and relevant dates, including periods of recruitment, exposure, follow-up, and data collection	3-4
Participants	6	(*a*) *Cohort study*—Give the eligibility criteria, and the sources and methods of selection of participants. Describe methods of follow-up<br>*Case-control study*—Give the eligibility criteria, and the sources and methods of case ascertainment and control selection. Give the rationale for the choice of cases and controls<br>*Cross-sectional study*—Give the eligibility criteria, and the sources and methods of selection of participants	3-4
		(*b*) *Cohort study*—For matched studies, give matching criteria and number of exposed and unexposed<br>*Case-control study*—For matched studies, give matching criteria and the number of controls per case	3-4
Variables	7	Clearly define all outcomes, exposures, predictors, potential confounders, and effect modifiers. Give diagnostic criteria, if applicable	3-4
Data sources/ measurement	8*	For each variable of interest, give sources of data and details of methods of assessment (measurement). Describe comparability of assessment methods if there is more than one group	*3-4*
Bias	9	Describe any efforts to address potential sources of bias	3-4
Study size	10	Explain how the study size was arrived at	5

Quantitative variables	11	Explain how quantitative variables were handled in the analyses. If applicable, describe which groupings were chosen and why	3-4
Statistical methods	12	(*a*) Describe all statistical methods, including those used to control for confounding	5
		(*b*) Describe any methods used to examine subgroups and interactions	5
		(*c*) Explain how missing data were addressed	5
		(*d*) *Cohort study*—If applicable, explain how loss to follow-up was addressed<br>*Case-control study*—If applicable, explain how matching of cases and controls was addressed<br>*Cross-sectional study*—If applicable, describe analytical methods taking account of sampling strategy	5
		(*e*) Describe any sensitivity analyses	5

Continued on next page

Results

Participants	13*	(a) Report numbers of individuals at each stage of study—eg numbers potentially eligible, examined for eligibility, confirmed eligible, included in the study, completing follow-up, and analysed	5-6
		(b) Give reasons for non-participation at each stage	5-6
		(c) Consider use of a flow diagram	5-6
Descriptive data	14*	(a) Give characteristics of study participants (eg demographic, clinical, social) and information on exposures and potential confounders	5-6
		(b) Indicate number of participants with missing data for each variable of interest	5-6
		(c) *Cohort study*—Summarise follow-up time (eg, average and total amount)	5-6
Outcome data	15*	*Cohort study*—Report numbers of outcome events or summary measures over time	5-6
		Case-control study—Report numbers in each exposure category, or summary measures of exposure	5-6
		Cross-sectional study—Report numbers of outcome events or summary measures	5-6
Main results	16	(*a*) Give unadjusted estimates and, if applicable, confounder-adjusted estimates and their precision (eg, 95% confidence interval). Make clear which confounders were adjusted for and why they were included	6-7-8
		(*b*) Report category boundaries when continuous variables were categorized	6-7-8
		(*c*) If relevant, consider translating estimates of relative risk into absolute risk for a meaningful time period	6-7-8
Other analyses	17	Report other analyses done—eg analyses of subgroups and interactions, and sensitivity analyses	6-7-8

Discussion

Key results	18	Summarise key results with reference to study objectives	8-9-10
Limitations	19	Discuss limitations of the study, taking into account sources of potential bias or imprecision. Discuss both direction and magnitude of any potential bias	10
Interpretation	20	Give a cautious overall interpretation of results considering objectives, limitations, multiplicity of analyses, results from similar studies, and other relevant evidence	8-9-10
Generalisability	21	Discuss the generalisability (external validity) of the study results	8-9-10

Other information

Funding	22	Give the source of funding and the role of the funders for the present study and, if applicable, for the original study on which the present article is based	10

*Give information separately for cases and controls in case-control studies and, if applicable, for exposed and unexposed groups in cohort and cross-sectional studies.

Note: An Explanation and Elaboration article discusses each checklist item and gives methodological background and published examples of transparent reporting. The STROBE checklist is best used in conjunction with this article (freely available on the Web sites of PLoS Medicine at http://www.plosmedicine.org/, Annals of Internal Medicine at http://www.annals.org/, and Epidemiology at http://www.epidem.com/). Information on the STROBE Initiative is available at www.strobe-statement.org.

References

1. Collins GS, Reitsma JB, Altman DG, Moons KGM. Transparent reporting of a multivariable prediction model for individual prognosis or diagnosis (TRIPOD): the TRIPOD statement. BMJ 2015; 350: g7594.

2. Martín-Rodríguez, F., Sanz-García, A., Del Pozo Vegas, C., Ortega, G. J., Castro Villamor, M. A., & López-Izquierdo, R. (2021). Time for a prehospital-modified sequential organ failure assessment score: An ambulance-Based cohort study. The American journal of emergency medicine, 49, 331–337.

3. Robin X, Turck N, Hainard A, et al. pROC: an open-source package for R and S+ to analyze and compare ROC curves. BMC Bioinformatics 2011; 12: 77.

4. Harrell Jr FE. rms: Regression Modeling Strategies. 2019.

5. Alba AC, Agoritsas T, Walsh M, et al. Discrimination and Calibration of Clinical Prediction Models: Users' Guides to the Medical Literature. JAMA. 2017 Oct 10;318(14):1377-1384.

6. Vickers AJ, Elkin EB. Decision curve analysis: a novel method for evaluating prediction models. Medical Decision Making. 2006 Nov-Dec;26(6):565-74.

7. https://www.mskcc.org/departments/epidemiology-biostatistics/biostatistics/decision-curve-analysis
